# Inhibition of *Listeria monocytogenes* ATCC 19115 on ham steak by tea bioactive compounds incorporated into chitosan-coated plastic films

**DOI:** 10.1186/1752-153X-6-74

**Published:** 2012-07-28

**Authors:** Dan C Vodnar

**Affiliations:** 1 Food Science and Technology Department, Unit of Chemistry and Biochemistry, University of Agricultural Sciences and Veterinary Medicine, 3-5 Mănăştur str, Cluj-Napoca, 400372, România

## Abstract

**Background:**

The consumer demands for better quality and safety of food products have given rise to the development and implementation of edible films. The use of antimicrobial films can be a promising tool for controlling *L. monocytogenes* on ready to eat products. The aim of this study was to develop effective antimicrobial films incorporating bioactive compounds from green and black teas into chitosan, for controlling *L. monocytogenes* ATCC 19115 on vacuum-packaged ham steak. The effectiveness of these antimicrobial films was evaluated at room temperature (20°C) for 10 days and at refrigerated temperature (4°C) for 8 weeks.

**Results:**

The HPLC results clearly show that relative concentrations of catechins and caffeine in green tea ranked EGCG>EGC>CAF>ECG>EC>C while in black tea extracts ranked CAF>EGCG>ECG>EGC>EC>C. The chitosan-coated plastic films incorporating green tea and black tea extracts shows specific markers identified by FTIR. Incorporating natural extracts into chitosan showed that the growth of *L monocytogenes* ATCC 19115 was inhibited. The efficacy of antimicrobial effect of tea extracts incorporated into chitosan-coated plastic film was dose dependent. However, chitosan-coated films without addition of tea extracts did not inhibit the growth of *L. monocytogenes* ATCC 19115*.* Chitosan-coated plastic films incorporating 4% Green tea extract was the most effective antimicrobial, reducing the initial counts from 3.2 to 2.65 log CFU/cm^2^ during room temperature storage and from 3.2 to 1–1.5 log CFU/cm^2^ during refrigerated storage.

**Conclusions:**

Incorporation of tea extracts into the chitosan-coated films considerably enhanced their effectiveness against *L. monocytogenes* ATCC 19115. 4% Green tea incorporated into chitosan-coated plastic film had a better antilisterial effect than 2% green tea or 2% and 4% black tea. Data from this study would provide new formulation options for developing antimicrobial packaging films using tea extracts to improve the microbiological safety and quality of ham steak during room and refrigerated storage.

## Background

*Listeria monocytogenes* is a foodborne pathogen in ready to eat products because its ability to survive and grow at refrigeration conditions, its capacity to tolerate relatively high heat and high concentrations of salt [1]. Consuming foods contaminated with *L. monocytogenes* causes the disease listeriosis, which affect especially elderly adults and adults with compromised immune systems and can cause meningitis [2]. The consumer demands for better quality and safety of food products have given rise to the development and implementation of edible films. The use of antimicrobial films can be a promising tool for controlling *L. monocytogenes* on ready to eat products [3].

Chitosan, derived by the diacetylation of chitin, is a major component of the shells of crustaceans [4]. Chitosan is a natural polymer, which is non-toxic, biodegradable and biocompatible and has intrinsic antimicrobial activity; however, chitosan film alone had no inhibitory effect on the growth of *L. monocytogenes* when applied to the surface of ham steaks [1]. Surface application of antimicrobials represents an alternative to direct application and is based on the incorporation of antimicrobial compound into an edible coating that is then applied onto the food [5]. The choice of active agents that could be incorporated into edible films could influence the consumers perception on ingredients listed on the product label [6]. Thus, a new alternative, by incorporating of natural bioactive compounds into edible films is needed.

After water, tea is one of the most consumed beverages in the world due to the bioactive compounds associated with numerous health benefits [7,8]. Studies reports on the benefits of green tea consumption, antioxidant and antimicrobial activity [7]. Obtain from the plant *Camellia sinensis*, green tea contains higher amounts of bioactive compounds indicating that catechins are the main representatives polyphenols with a wide range of functional and antimicrobial activities [9]. The most important catechins are catechin, epicatechin, epicatechin gallate, epigallocatechin, epigallocatechin gallate [10]. Some studies show that green tea extracts act as inhibiters of food pathogens including *Staphylococcus aureusShigella disenteriaeVibrio choleraeCampylobacter jejuni and Listeria monocytogenes*[11]. In black tea, the major polyphenols are tearubigines and theaflavines [12]. The antimicrobial activity of black tea has been also reported [13].

The overall objective of this study was to develop effective antimicrobial films incorporating bioactive compounds from green and black teas, for controlling *L. monocytogenes* ATCC 19115 on vacuum-packaged ham stake. The effectiveness of these antimicrobial films was evaluated at room temperature (20°C) for 10 days and at refrigerated temperature (4°C) for 8 weeks.

## Results and discussion

### HPLC characterization of tea extracts

**Figure 1:**
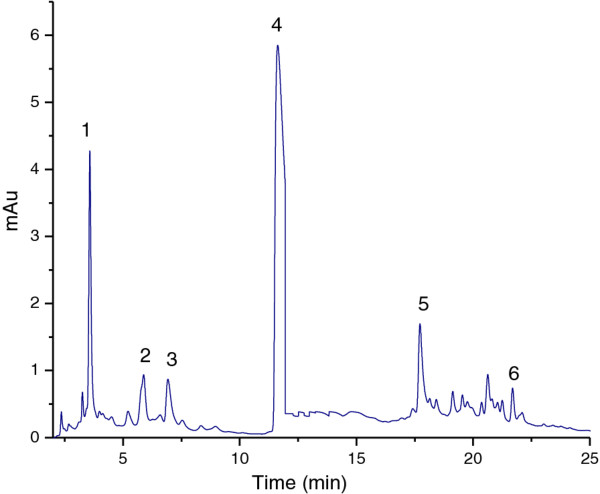
**HPLC chromatogram of tea polyphenols.** The peak numbers indicate: 1, EGC, t_R_ = 3.6; 2, C, t_R_ = 5.81; 3, EC, t_R_ = 6.99; 4, EGCG, t_R_ = 11.71; 5, CAF, t_R_ = 17.73 and 6, ECG, t_R_ = 21.67.

HPLC analysis of catechins and caffeine contained in green and black teas was performed. Based on the retention times of the peaks from the chromatograms (Figure 1), each peaks of 2 samples, tea extracts and authentic standards, were compared. The concentration of catechins and caffeine in the green tea and black tea extracts are presented in Table 1. There was a variation in the concentration of catechins between the green and black tea extracts analyzed. The green tea extract showed higher amounts in all investigated catechins and caffeine then black tea extract. Considerable variation in the levels of catechins has been reported in several studies [14,15]. Comparing the levels of catechins presented in Table 1, relative concentrations of catechins and caffeine in green tea ranked EGCG > EGC > CAF > ECG > EC > C while in black tea extracts ranked CAF > EGCG > ECG > EGC > EC > C. Some studies found the following relative concentration of catechins: EGCG > ECG > EC > C [15]. The results found for the green tea extracts followed this same sequence reported also by Saito et al. [16]. With regard to caffeine, the black tea extract had a higher concentration than green tea as showed in table 1. In addition, it can be noted that the level of catechins and caffeine in green tea and black tea extracts increased once with their concentrations.

**Table 1:** Main concentration (mg%) of catechins and caffeine in green (GT) and black teas (BT) extracts

**Signal**	**Compound**	**2% GT**	**4% GT**	**2% BT**	**4% BT**
1.	EGC	204.2 ± 4.2	408.5 ± 8.9	18.9 ± 0.4	36.6 ± 0.3
2.	C	9.4 ± 0.4	18.6 ± 5.2	7.5 ± 0.1	15.0 ± 0.2
3.	EC	13.1 ± 0.1	26.4 ± 0.5	10 ± 0.1	20 ± 0.3
4.	EGCG	240.8 ± 3.6	481.6 ± 6.2	54.0 ± 1	109.8 ± 1.4
5.	CAF	103.6 ± 1.8	207.4 ± 3.7	89.6 ± 0.8	178.5 ± 2.1
6.	ECG	67.8 ± 1.4	131.4 ± 1.6	43.6 ± 0.8	86.5 ± 1.6

### FTIR spectroscopy

**Figure 2:**
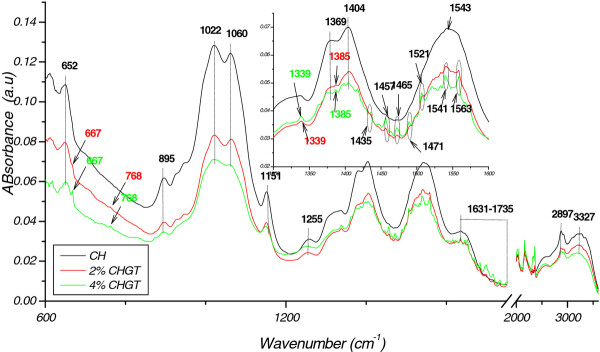
FTIR spectra of chitosan film (CH), Chitosan incorporating 2% (CHGT2%) and 4% (CHGT4%) green tea extract.

FTIR spectroscopy is expected to be especially valuable in analyzing the phase structure and the interaction between chitosan and green tea extracts. The infrared spectra of chitosan films with and without addition of 2% or 4% green tea extracts and their blends are shown in Figure 2. Stretching vibration of hydroxyl groups (−OH) appeared around 3327 cm^-1^ in all samples and indicated intermolecular bonding of chitosan molecules. The peak at 2897 cm^-1^, corresponded to C-H stretching, while the band at 1336 cm^-1^ was the O-H of water. The chitosan film spectrum was very similar to that reported by Bourtoom et al. [17]. The region between 1631–1735 cm^-1^ corresponded to C = O stretching (amide I) and the peak at 895 cm^-1^ suggested the presence of an ether group in the film. The chemical interactions are reflected by changes in peaks of characteristics spectra after blending the chitosan with tea extracts. The characteristic peaks for green tea and black tea (not shown) appeared as a small shoulder at 667, 768, and in the region between 1339–1563 cm^-1^ specify for C = O-NHR, amine, -NH_2_, and ammonium. Spectra showed a peck near 1400 cm^-1^, which was the same pattern as reported by Vodnar et al. [18].

### Effect of active films on the growth of *L. monocytogenes* ATCC 19115 on ham steaks at room temperature storage

The antimicrobial activity of chitosan has been showed in many studies [19,20] but antimicrobial proprieties may become negligible when it is use in the form of insoluble films [21]. Several studies showed the antimicrobial effect of commercial substances such as nisin, sodium lactate [21,22], but have disadvantages, nisin is relatively expensive and the utilization in high levels would be prohibited for the food industry, sodium lactate needs to be incorporated into high concentration to have antimicrobial properties. Additionally, the utilization of high levels of antimicrobials can have a negative impact on the customer perceptions.

**Figure 3:**
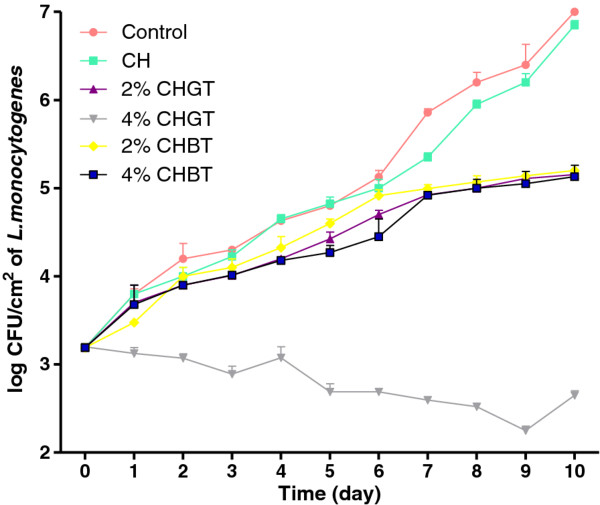
**Effect of chitosan-coated plastic films (CH) incorporating 2%, 4% Green tea (CHGT) and 2%, 4% Black tea (CHBT) extracts on the growth of *L. monocytogenes* on ham steak at room temperature storage.**

The effect of tea extracts incorporated into chitosan-coated plastic films on the growth of *L. monocytogenes* ATCC 19115 on ham steaks is shown in Figure 3. The initial concentration of *L. monocytogenes* ATCC 19115 on inoculated ham steak samples was 3.2 log CFU/cm^2^. *L. monocytogenes* ATCC 19115 in chitosan-coated plastic films grew to 6.8 log CFU/cm^2^ after 10 days of storage at room temperature. In control ham steak sample, the count reached 6.9 log CFU/cm^2^ after 10 days, demonstrating the ability for the pathogen to grow rapidly in package without tea extracts. Incorporating natural extracts into chitosan showed that the growth of *L. monocytogenes* ATCC 19115 was inhibited. 4% CHGT was the most effective antimicrobial reducing the initial counts from 3.2 to 2.65 log CFU/cm^2^ after 10 days of room storage. The counts of *L. monocytogenes* ATCC 19115 in samples 2% CHGT, 2% CHBT, 4% CHBT were not statistically significant (p < 0.05). In all samples the growth of *L. monocytogenes* ATCC 19115 is progressive from 1 to 10 days, exception sample 4% CHBT where is registered a constant inhibition of pathogen. The efficacy of antimicrobial effect of tea extracts incorporated into chitosan-coated plastic film was dose dependent. The 2% green tea and 2%, 4% black tea extracts had inhibitory effect on *L .monocytogenes* ATCC 19115 but the most efficient antimicrobial effect has been registered by 4% CHGT. The chitosan-coated plastic film (CH) and the control samples showed a very similar growth rate of *L. monocytogenes* ATCC 19115. A possible explanation is that chitosan is ineffective in films because has a low rate of diffusion in a compact matrix like ham steak [21]. Compare with chitosan the incorporated tea extracts had varying efficacy but all of them are able to migrate from the chitosan film to ham steak. The concentration of tea extracts, the interaction with food components and the diffusion rate of them could signify the antimicrobial activity of biofilms.

### Effect of active films on the growth of *L. monocytogenes* ATCC 19115 on ham steaks during refrigerated storage

**Figure 4:**
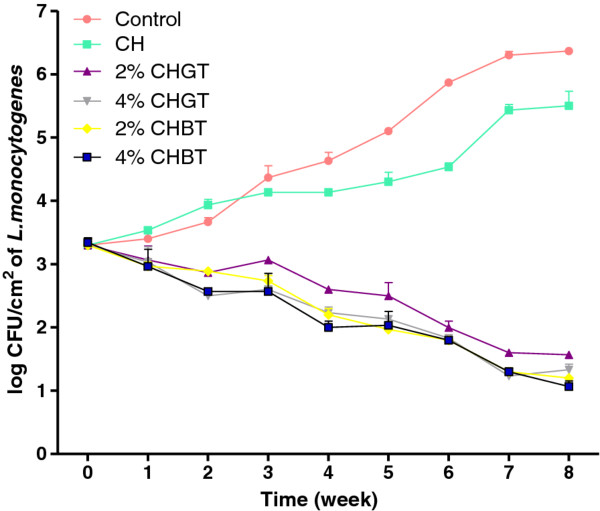
**Effect of chitosan-coated plastic films (CH) incorporating 2%, 4% Green tea (CHGT) and 2%, 4% Black tea (CHBT) extracts on the growth of *L. monocytogenes* on ham steak stored at 4°C.**

Inoculated ham steak samples with an initial population of 3.2 log CFU/cm^2^, were stored at 4°C for 8 weeks. Counts of *L. monocytogenes* ATCC 19115 on ham steak samples treated with chitosan-coated film incorporating tea extracts are shown in Figure 4. In the first 3 weeks, *L. monocytogenes* ATCC 19115 in control and chitosan-coated plastic film grew very slowly from 3.2 to 4.1 log CFU/cm^2^. It started to growth rapidly for week 3 and reached 6.3 log CFU/cm^2^ for control sample and 5.5log CFU/cm^2^ for CH sample after 8 weeks storage at 4°C. The tea extracts addition into chitosan-coated films were effective against *L monocytogenes* ATCC 19115 and reduced its counts from 3.2 to 1–1.5 log CFU/cm^2^ during 8 weeks of storage. The treatment with 4% CHGT was the most effective and completely inhibited the growth of *L. monocytogenes* ATCC 19115 after 8 weeks storage of 4°C, decreasing from 3.2 to 1 log CFU/cm^2^. These results demonstrate that chitosan-coated plastic films incorporated with 4% CHGT could be use to control *L. monocytogenes* ATCC 19115 on ham steaks. Several studies have shown that the polyphenolic components in green and black teas effectively inhibit the growth of *S. mutans* and S*. sobrinus*[23,24]. Almajano et al. [25] found that the strain *B. cereus* is a very sensitive bacterium showing the largest inhibition rate in the presence of tea extracts. In general the gram negative bacteria are more resistant to polyphenols than Gram positive bacteria, perhaps due to the different cell wall compositions [26].

Others have studied the decline of *L. monocytogenes* on low-moisture ready-to eat meat products. *L monocytogenes* declined on vacuum-packaged beef snack sticks by 1 log CFU/sample in 5 days [27]. In inoculated pepperoni and vacuum-packed *L. monocytogenes* decreased >1.5 > 2.5 and >3 log CFU/cm^2^ on samples stored at 4, 12, or 25°C, respectively [28]. Chitosan is a polymer that imparts minimal adverse sensory properties to food [1]. The quality properties of pork sausage prepared with water-soluble chitosan has been investigated and no differences in colour flavour, texture, overall acceptance and mechanical texture was detected by Jo et al. [29]. As such, this study assume that green tea and black tea extracts and chitosan at the levels described, would not affect the organoleptic properties of ham steaks and the results cannot be extrapolated to the species as a whole without further work.

The antilisterial activity was higher in chitosan-coated plastic film incorporating 4% green tea. These results demonstrated that the antimicrobial films developed in this study could potentially be used to extend the microbial shelf life of ham steak.

## Conclusions

The results of this study clearly show that relative concentrations of catechins and caffeine in green tea ranked EGCG > EGC > CAF > ECG > EC > C while in black tea extracts ranked CAF > EGCG > ECG > EGC > EC > C. The FTIR spectra indicated that interactions and molecular miscibility were present between the major components of green tea, black tea extracts and chitosan.

The effect of chitosan-coated plastic film incorporating green tea and black tea extracts on the growth of *L. monocytogenes* ATCC 19115 in ham steak was investigated during room and refrigerated storage. However, chitosan-coated films without addition of tea extracts did not inhibit the growth of *L. monocytogenes* ATCC 19115. Incorporation of tea extracts into the chitosan-coated plastic films considerably enhanced their effectiveness against *L. monocytogenes* ATCC 19115. 4% Green tea incorporating into chitosan-coated film had a better antilisterial effect than 2% green tea, 2%, 4% black tea.

Data from this study would provide new formulation options for developing antimicrobial packaging films using tea extracts to improve the microbiological safety and quality of ham steak during room and refrigerated storage.

## Methods

### Preparation of tea extracts

Green tea (GT) and Black tea (BT) were purchased from a Romanian market. The aqueous extracts were made by adding 100 ml water (100°C) to 1 g or 2 g tealeaves and brewing for 10 min with stirring and removing solid matter by filtration.

### Chemical characterization of tea extracts

A HPLC Agilent Technologies 1200 Series chromatograph coupled with UV–VIS detector and an HPLC column Supelcosil LC-18 column (Sigma-Aldrich Co), 5 μm, 25 cm x 4.6 mm was used. The gradient elution was performed with mobile phase A, composed of methanol: acetic acid: double distilled water (10:2:88 v/v/v) and mobile phase B, comprising methanol: acetic acid: double distilled water (90:3:7 v/v/v), at a flow rate of 1.0 ml/min. All solvents were HPLC grade solvents, filtered through a 0.45-*μ*M membrane (Millipore, U.S.A.) and degassed in an ultrasonic bath before use. The chromatograms were monitored at 280 nm.

### Culture growth conditions

*L. monocytogenes* ATCC 19115 was maintained on Oxford agar (Sifin, Germany) agar plates at 4°C. For growth, a single colony of *L. monocytogenes* was inoculated into a tube of tryptic soy broth plus 0.7% yeast extract (TSBYE) (Difco Laboratories) and incubated at 35°C for 24 h. The culture was then transferred to 10 ml of fresh and incubated for 24 h at 35°C for 24 h to reach a final concentration of 10^9^ CFU/ml.

### Preparation of antimicrobial films

Two grams of low molecular weight (LMW) chitosan (Sigma Aldrich, Germany) were dissolved in 100 ml of 1% (w/v) acetic acid and stirred overnight at room temperature. Hydroxypropyl methylcellulose (HPMC) (Sigma-Aldrich) solution was prepared by dissolving 3 g of HPMC in 100 ml of 1% acetic acid. The coating solution was prepared by mixing 12 parts of chitosan solution with 2 parts of HPMC solution and 1 part of tea extract. A Surlyn® film (1-mil) was taped into 20x20 cm glass plates and 15 ml of chitosan solution was cast onto the plastic film using a thin-layer chromatography plate coater (TLC, Switzerland). The gate of the TLC coater was fixed at 500 μm to control the thickness of the coating. The chitosan-coated film incorporating 1%, 2% green tea and black tea extracts. The coated films were air-dried at room temperature overnight.

### FTIR characterization of antimictobial films

FTIR spectra using attenuated total reflectance (ATR) and an internal reflection accessory made of composite zinc selenide (ZnSe) and diamond crystals were obtained on a Schimatzu IR Prestige- 21 spectrometer. Each spectrum was registered from 4000 to 500 cm^-1^. The FTIR spectra were recorded for all samples in parallel with controls. Three spectra were acquired for each trial variant at room temperature. Each spectrum was composed of an average of 128 separate scans. The measuring time was approximately 9 minutes per sample (n = 3), depending on the number of scans per spectrum. Accordingly, as the average number of scans increased, the measuring time increased.

### Inoculation of ham steak

Freshly processed ham steak samples were obtained from a local retailer. They were kept frozen at −20°C and thawed at 2°C for 1 day immediately before use as described by Besse et al. [30]. Slices of ham steak were punched aseptically into 6.2 cm diameter round pieces weighing 26.4 g with a surface area of 25.9 cm^2^ on one side. The ham steak discs were placed onto a piece of sterile aluminium foil, 125 μl of *L. monocytogenes* ATCC 19115 was spread on one side of the ham steak surfaces, and the sample were left undisturbed for 5 min to allow the inoculum to soak in and the cells to attach. The ham steak discs were then flipped and the same procedure was repeated for the other side of each sample thus achieving the final concentration of 2x 10^3^ CFU/cm^2^ of ham steak surface. After inoculation, ham steak discs were kept at 4°C for 20 minutes to allow bacterial attachment.

### Packaging of inoculated ham steak samples with antimicrobial films

The inoculated samples were wrapped in the antimicrobial and control films prepared as described above. The wrapped samples were then inserted into 3-mm thick high barrier pouches (nylon/polyethylene, Koch Supplies) and subsequently sealed using vacuum-packaging machine **(Bag** Unit, Zepter). The packages were stored at 20°C for 10 days. Instead of using 4°C, a typical refrigerated storage temperature for this kind of products, a relative high temperature was used to accelerate the selection of the most effective antimicrobial concentration of green and black tea against *L. monocytogenes* ATCC 19115*.* The packages prepared for refrigeration storage (4°C, 8 weeks) were analyzed weekly.

### Analysis of *L. monocytogenes* in the samples

The samples were analyzed for *L. monocytogenes* ATCC 19115 every 2 days over the storage period. For determination of *L. monocytogenes* ATCC 19115 counts, a package was opened aseptically and contents were transferred to a sterile stomacher bag and homogenized for 2 min with 100 ml of 0.9% NaCl water. Counts of *L. monocytogenes* ATCC 19115 were determined by an overlay method [31]. The plates were incubated at 35°C for 48 h and black colonies were counted.

### Statistical analysis

Results for 3 individual experiments were used to calculate the mean of cell counts. Analysis of variance (ANOVA) and Duncan’s multiple range tests were performed to analyze the results. Significance of difference was defined at the 5% level (P < 0.05). All statistical analysis was carried out using Graph Pad Version 4.0 (Graph Pad Software Inc; San Diego, CA, USA).

## Abbreviations

BT, Black tea; C, Catechin; CAF, Caffeine; CH, Chitosan-Coated plastic film; CHBT, Chitosan-coated plastic film incorporating Black Tea extract; CHGT, Chitosan-coated plastic film incorporating Green Tea extract; EC, Epicatechin; ECG, Epicatechin Gallate; EGC, Epigallocatechin; EGCG, Epigallocatechin Gallate; GT, Green Tea.

## Competing interests

The author declares no competing interests.

## Authors' contributions

DCV carried out all experiments and prepared the final manuscript.
